# Perceived safety and trust in SAE Level 2 partially automated cars: Results from an online questionnaire

**DOI:** 10.1371/journal.pone.0260953

**Published:** 2021-12-21

**Authors:** Sina Nordhoff, Jork Stapel, Xiaolin He, Alexandre Gentner, Riender Happee

**Affiliations:** 1 Department Transport & Planning, Delft University of Technology, Delft, The Netherlands; 2 Department Cognitive Robotics, Delft University of Technology, Delft, The Netherlands; 3 Toyota Motor Europe NV / SA, Zaventem, Belgium; Universitat de Valencia, SPAIN

## Abstract

The present online study surveyed drivers of SAE Level 2 partially automated cars on automation use and attitudes towards automation. Respondents reported high levels of trust in their partially automated cars to maintain speed and distance to the car ahead (***M*** = 4.41), and to feel safe most of the time (***M*** = 4.22) on a scale from 1 to 5. Respondents indicated to always know when the car is in partially automated driving mode (***M*** *=* 4.42), and to monitor the performance of their car most of the time (***M*** = 4.34). A low rating was obtained for engaging in other activities while driving the partially automated car (***M*** *=* 2.27). Partial automation did, however, increase reported engagement in secondary tasks that are already performed during manual driving (i.e., the proportion of respondents reporting to observe the landscape, use the phone for texting, navigation, music selection and calls, and eat during partially automated driving was higher in comparison to manual driving). Unsafe behaviour was rare with 1% of respondents indicating to rarely monitor the road, and another 1% to sleep during partially automated driving. Structural equation modeling revealed a strong, positive relationship between perceived safety and trust (*β* = 0.69, *p* = 0.001). Performance expectancy had the strongest effects on automation use, followed by driver engagement, trust, and non-driving related task engagement. Perceived safety interacted with automation use through trust. We recommend future research to evaluate the development of perceived safety and trust in time, and revisit the influence of driver engagement and non-driving related task engagement, which emerged as new constructs related to trust in partial automation.

## 1. Introduction

Since the early 19th century, we trust cars driving at high speeds in complex traffic. However, vehicle automation technology has not yet reached a level of maturity comparable to steering systems, brakes and powertrains underlying our trust in manually driven cars. A range of today’s passenger cars provides SAE Level 2 automation through the combination of Adaptive Cruise Control (ACC) and Lane Keeping Assistance (LKA). These SAE Level 2 systems require the permanent supervision of human drivers to ensure the reliable and safe operation of the automated driving system. Driver monitoring systems have already been implemented in passenger cars since the early 2000s. Daimler equipped their Mercedes Benz model series with drowsiness detection algorithms using the steering wheel behavior of drivers. Further monitoring technologies include infrared eye-tracking systems and heart rate activity monitors [1–3]. These technologies monitor driver’s readiness to take over control from the automated car on highways, allowing drivers to perform non-driving related tasks for a few seconds.

The reliable and safe use of SAE Level 2 systems has been questioned in the context of the severe accidents with Tesla’s Autopilot system. Studies have pointed to automation misuse (e.g., prolonged hands-free driving, ignoring warnings to place hands back on the steering wheel, testing the limits of the operational design domain, mode confusion, engagement in secondary activities, using the system in bad weather conditions) [4–8]. These safety-critical behaviors may have been induced by the marketing and media, exaggerating the capabilities of automated vehicles and their expected market release [9–14]. A second plausible explanation pertains to the nature of partial automation. In partially automated cars, drivers have to constantly monitor the performance of the automated driving system in terms of its ability to control steering, acceleration and braking, and reacting to objects and events in the external environment [15]. Research has shown that humans have difficulties to monitor systems over extended periods of time and develop over-trust in automation if the systems show reliable and consistent behaviour, and engage in secondary activities, even when they are explicitly instructed to monitor the automation. This hampers the ability to safely take over control [16–19].

Calibrating trust (i.e., aligning trust with the actual trustworthiness or capabilities of the system) has been frequently discussed in this context in order to ensure an appropriate use of automation [5, 6, 20–24]. Trust is a multidimensional psychological concept that has received numerous conceptualizations and operationalizations [6, 25–28]. Perhaps the most cited (26,472 times, October 2021) definition of trust is *“the willingness of a party to be vulnerable to the actions of another party based on the expectation that the other party will perform a particular action important to the truster*, *irrespective of the ability to monitor or control that other party”* [26, p. 712]. Trust has been identified as a predictor of (automated driving) technology acceptance [8, 29–33].

The factors influencing trust in automation relate to the individual using the system, the environment, and the system [18]. The trust variability model [31] identified thirty-six generalizable factors of trust in automation, merging these into main factors such as initial learned trust (pre-existing knowledge; e.g., attitudes / expectations, brand, reputation, experience), dynamic learned trust (system performance, e.g., system reliability, validity, predictability), design features (e.g., ease of use, level of control, communication style, appearance), as well as internal context factors (e.g., age, gender). Trust in automation has also been associated with secondary task engagement and comfort. It is postulated that *“in order to achieve optimal task performance*, *drivers must be comfortable relying on the vehicle automation to drive so that they can effectively engage in a secondary task”* [33, p. 2]. The authors found that trust had strong positive effects on secondary task performance when the respondent’s situation awareness was high [33]. Studies have further shown that robust and reliable automation increases people’s trust and consequently the willingness to divert attention from the road and engage in secondary activities [34, 35].

The passive nature of partial automation can reduce driver engagement. Current partially automated cars require that users keep at least one hand on the wheel. This requirement increased driver engagement [8]. Driver engagement was evaluated based on whether systems provided information to drivers about the status of the system (i.e., clearly indicating whether the system is engaged or disengaged) using visual, audible, or haptic signals or a combination of these; monitor or see the driver to determine the level of engagement; and collaborate with drivers by giving them the option of full control and working with and not against their intention (i.e., the system should stay engaged but should always be overridable) [36].

Perceived safety is related to trust and has been identified as a basic human need and key predictor of automated vehicle acceptance [37, 38]. Similar to trust, perceived safety has received numerous definitions. Perceived safety was contrasted with actual safety: It is the *“feeling of security because people will leave their welfare directly to a technical machine that is not working transparent”* [39, p. 20]. In other studies, perceived safety was defined as *“the degree to which an individual believes that using automated vehicles will affect his or her well-being”* [40, p. 55], as *“a climate in which drivers and passengers can feel relaxed*, *safe and comfortable*, *while driving”* [38, p. 323], or as the condition of being secure from accidental harm, distinguishing it from intentional harm [41]. The perception of safety varies strongly between individuals and depends on context and past experience [23, 42, 43]. Perceived safety is closely related to objective safety [44, 45], but also depends on motives [46], and is thus inherently subjective. It can be influenced by the vehicle’s “personality” [47], aesthetics of an interface [48], information [21], and sound related to perceived safety in public spaces (i.e., sound affected perceived safety in public places) [49].

Several studies have modelled the relationship between trust and perceived safety in automated driving. Trust was modelled as predictor of perceived safety, while in other studies perceived safety was modelled as predictor of trust. In the study of [50], trust was modelled as a function of perceived safety and privacy risk, perceived ease of use and usefulness. Furthermore, trust influenced the attitude towards using automated cars. In the model of [38], trust served as predictor of perceived usefulness, ease of use, perceived safety, behavioral intention, and willingness to re-ride automated cars. Perceived usefulness, trust, and perceived safety predicted the intention to use automated cars. The perceived benefits of automated cars (commonly measured by the technology acceptance constructs perceived usefulness / performance expectancy) influenced acceptance [38, 50, 51]. Perceived safety was modelled as direct predictor of trust, while trust served as direct predictor of acceptance [52]. We model trust as a function of perceived safety. As the literature has revealed effects of both trust and perceived safety on acceptance, we hypothesize that perceived safety and trust are both positive predictors of acceptance. Furthermore, we posit that driver engagement, non-driving related task engagement and the perceived benefits of partially automated driving influence acceptance.

The present study addresses the following research questions, using a new online survey targeting drivers of partially automated cars.

*Research question 1*: *What are the activities that drivers of partially automated cars engage in during manual and partially automated driving*?

*Research question 2*: *How are the perceived safety and trust in partially automated cars operationalized*?

*Research question 3*: *To what extent do drivers perceive their partially automated cars safe and trustworthy*?

*Research question 4*: *How are the perceived safety and trust in partially automated cars related*?

*Research question 5*: *How are perceived safety*, *trust*, *perceived benefits*, *engagement in driving and non-driving related tasks related with the acceptance of partially automated cars*?

## 2. Method

### 2.1 Instrument and recruitment

The recruitment targeted current users of partially automated cars. We distributed the survey at Tesla’s supercharging stations near Utrecht, Dordrecht and Amsterdam in the Netherlands in the form of a QR code. The link was further distributed among members of Tesla Owners clubs [53] and Tesla Owners forums [54]. In order to target drivers of partially automated cars of other brands, the survey was distributed in car-and mobility-related forums and groups of Reddit and Facebook, respectively. The authors of the present study further shared the link to the questionnaire on LinkedIn. Further, an anonymous link to access the questionnaire was sent to employees of Toyota Motor Europe using internal communication mailing.

https://www.tesla.com/de_DE/support/ownersclubhttps://www.tff-ev.de/ An online questionnaire was created on Qualtrics.com [55]. Instructions informed the respondents that it would take around 20 minutes to complete the questionnaire and that the study is organized by Delft University of Technology in the Netherlands. In order to warrant data quality, Qualtrics applied a number of technologies that ensured that respondents did not take the survey more than once, that suspicious, non-human (i.e., bot) responses were detected, and that search engines were prevented from indexing the survey.

### 2.2 Questionnaire content

#### 2.2.1 System functionality and provision of written consent to participate in study

Prior to participation in the questionnaire, respondents received a description about the functionality of partially automated cars in order to ensure that respondents had a sufficient understanding of partially automated cars.

*Have you* ***heard of partly automated cars***? *With this questionnaire*, *we would like to get* ***your opinion on partly automated cars*** *which are already commercially available*. ***Partly automated cars automate the acceleration*, *braking*, *and/or steering of the car***. *This implies that they* ***control the speed and distance to the car in front and/or the steering*, *keeping the car in the lane***. *They* ***have gas and brake pedals and a steering wheel***. *When the car is driving in partly automated mode*, ***you as driver have to supervise the performance of the car in order to continue manual driving***. *Your* ***hands have to remain on the steering wheel*, *or alternatively*, *you have to periodically touch the steering wheel***. *Your* ***eyes remain on the road***.

After the respondents received the instructions, they were asked to provide their written consent to participate in the study. They were asked to declare that they had been informed in a clear manner about the nature and method of the research as described in the instructions at the beginning of the questionnaire. They were further asked to agree, fully and voluntarily, to participate in this study. They were further informed that they retain the right to withdraw their consent and that they can stop participation in the study at any time. Finally, they were informed that their data will be treated anonymously in scientific publications, and will not be passed to third parties without their permission.

#### 2.2.2 Personal information

After having been asked to provide the written consent to participate in the study, respondents were asked to provide information about their socio-demographics (i.e., age, gender, education), personality, driving behavior and frequency of use of their partially automated cars (e.g., access to valid driver license, age, brand, and model of car, effect of COVID-19 on mileage, accident involvement). Respondents were asked to indicate their access to Lane Departure Warning (LDW), Lane Keeping Assist (LKA), and Adaptive Cruise Control (ACC) in their cars, and how often they activate those systems. Only respondents who indicated that they had access to all three systems (i.e., LDW, LKA, and ACC) or a combination of two of the three systems (i.e., LDW and LKA / ACC) were navigated to the questions that asked them to rate their attitudes towards and experiences with their partially automated cars. If they did not fulfil this condition, they were directed to the final questionnaire section on the evaluation of six Human Machine Interfaces that are adopted in commercially available in today’s passenger cars (Cadillac SuperCruise, Toyota Safety Sense 2.0, Tesla Autopilot).

#### 2.2.3 Attitudinal statements

Respondents were asked to indicate on a Likert scale from strongly disagree (1) to strongly agree (5) which types of behaviors they experienced with their partially automated cars. They were further asked to indicate on a scale from strongly disagree (1) to strongly agree (5) to what extent they trust their cars to perform partially automated driving manoeuvres (e.g., keeping the car centered in the lane, maintaining speed and distance to the car ahead). Further questions pertained to the behaviour of drivers in partially automated cars such as whether respondents felt hesitant to activate the partially automated driving mode from time to time, and whether they engaged in secondary activities. Respondents were also asked to indicate to what extent their partially automated car keeps them engaged in the driving task, and the frequency with which they engage in certain types of activities during manual and partially automated driving. Respondents were further asked for their motives to use their partially automated car, and the reasons for deactivating the system. Furthermore, respondents had to indicate to what extent they feel safe as a driver in their partially automated cars.

The order of these attitudinal questions was randomized in order to rule out order effects.

### 2.3 Analysis of responses

First, descriptive statistics (i.e., means, standard deviations, and frequencies) were calculated for the questionnaire items. Mean ratings were compared in order to identify the highest, moderate, and lowest mean ratings.

Second, a confirmatory factor analysis was performed to confirm the latent structure in the dataaset. The output of the confirmatory analysis is the measurement model, which assesses the measurement relationships between the latent (i.e., unobserved / hypothetical component or factor) and observed variables (i.e., questionnaire items). The psychometric properties of the measurement model were assessed by its indicator reliability, internal consistency reliability, convergent validity and discriminant validity. Convergent validity was assessed by four criteria: 1) All scale items should be significant and have loadings exceeding 0.60 on their respective scales, 2) the average variance extracted (AVE) should exceed 0.50 [3] construct reliability (CR) and 4) Cronbach’s alpha values should exceed 0.70 [56, 57]. Discriminant validity of our data was examined with the test of squared correlations by [56], which implies that the correlation coefficient between two latent variables should be smaller than the square root of the average variance extracted (AVE) of each latent variable to demonstrate sufficient discriminant validity.

The third step of the analysis involved testing the structural model. Maximum-likelihood (ML) estimation was used to estimate the measurement and structural model, which has proven robust to violations of the normality assumption [58].

The confirmatory factor analysis and structural equation modeling were performed with R software lavaan package [59].

### 2.4 Data filtering

In total, 1,557 questionnaires were completed. The data was collected between November 24, 2020 and January 30, 2021. On average, respondents needed 78.78 minutes to complete the survey (note that the responses were recorded after one week of the last activity of respondents). In order to enhance data quality, we applied a strict data screening: Respondents were excluded if they were identified as bots (*n* = 46), if they did not agree to participate in the study (*n* = 10), if they took an unreasonable amount of time to complete the survey (i.e., less than 2 and more than 9551 minutes) (*n* = 311), if they did not report to have access to a valid driver license (*n* = 14). *“I prefer not to respond”* and *“Not applicable to me”* responses were defined as missing values and excluded from the analysis. 1,137 responses remained for the analysis.

## 3. Results

### 3.1 Respondents

An overview of respondents’ profile is provided in Table 1.

**Table 1:** Overview of respondents’ profile after data filtering (M = mean, SD = standard deviation, relative frequencies, n = number of respondents).

Question	*M*	SD	Relative frequencies of response categories		*n*
Age (Q2)	3.53	0.80	Below 18 (1)	1%	1129
			18–22 (2)	9%	
			23–35 (3)	37%	
			36–55 (4)	45%	
			56–69 (5)	9%	
Gender (Q3)	1.20	0.42	Male (1)	80%	1137
			Female (2)	19%	
			Other (3)	1%	
Education (Q4)	3.45	1.02	High school diploma without apprenticeship / professional training (1)	8%	1115
			High school diploma with apprenticeship / professional training (2)	11%	
			College degree (3)	16%	
			Bachelor / Master degree (4)	59%	
			PhD / Dr degree (5)	6%	
Access to driver license (Q15)	1	0	Yes (1)	100%	1101
			No (0)	0%	
Age of car (Q16)	1.90	1.18	Less than 2 years (1)	52%	1097
			Less than 5 years (2)	23%	
			Between 5 and 10 years (3)	13%	
			Between 11 and 15 years (4)	5%	
			More than 15 years (5)	6%	
Car brand (Q17)	-	-	Tesla	35%	960
			Toyota	25%	
			BMW	5%	
			Volkswagen	5%	
			Ford	4%	
			Honda	4%	
			Lexus	3%	
			Audi	3%	
			Hyundai	3%	
			Mercedes Benz	3%	
			Nissan	2%	
			Volvo	2%	
			Peugeot	1%	
			Kia	1%	
			Chevrolet	1%	
			Renault	1%	
			Fiat	1%	
			Jeep	1%	
			Citroën	1%	

### 3.2 Ratings of attitudinal questions

Means, standard deviations, and relative frequencies are shown in Table 2, ordered from highest to lowest mean score.

**Table 2:** Descriptive statistics of attitudinal questions (M, SD, 1 = strongly disagree, 2 = disagree, 3 = neutral, 4 = agree, 5 = strongly agree, n = number of respondents). Means were ordered from highest to lowest in order to show high, moderate, and low mean ratings.

Question	*M*	SD	Relative frequencies of response categories						*n*
			1	2	3	4	5	6	
Q26. Please indicate how often you use your automated car with speed and steering support. * [60, 61]	4.70	1.44	3%	7%	12%	16%	20%	42%	698
Q35. I always know when my car is in partly automated driving mode. [62]	4.42	0.87	1%	4%	5%	30%	59%	–	645
Q31. I trust my partly automated car to maintain speed and distance to the car ahead. [63]	4.41	0.85	1%	4%	7%	31%	58%	–	641
Q46. I use my partly automated car because it helps me to reach my destination more comfortably. [38, 51]	4.38	0.90	2%	3%	7%	30%	57%	–	614
Q42. I monitor the performance of my partly automated car most of the time. **	4.34	0.87	2%	3%	7%	35%	53%	–	644
Q55. I feel safe most of the time. [38, 64, 65]	4.22	0.75	1%	2%	11%	49%	38%	–	613
Q45. I use my partly automated car because it helps me to reach my destination more safely. [38, 51]	4.17	0.93	2%	4%	13%	37%	44%	–	615
Q38. My partly automated car always reminds me to keep my hands on the steering wheel. **	4.15	1.17	7%	5%	8%	27%	53%	–	627
Q47. I use my partly automated car because it makes driving more pleasurable. **	4.09	1.08	3%	7%	12%	32%	46%	–	612
Q56. I feel relaxed most of the time. [38, 51]	4.07	0.85	1%	5%	13%	50%	32%	–	612
Q39. My partly automated car helps me to keep using it in the manner as advised by the manual. **	4.02	0.94	2%	5%	18%	40%	35%	–	630
Q40. I can trust my partly automated car. [29, 65]	3.97	0.97	2%	8%	13%	45%	32%	–	647
Q37. My partly automated car always reminds me to take back full control. **	3.92	1.20	6%	8%	17%	27%	42%	–	620
Q32. I trust my partly automated car to keep the car centered in the lane. [62]	3.89	1.13	4%	10%	14%	36%	36%	–	622
Q60. I entrust the safety of a close relative to my partly automated car. [66]	3.86	1.06	3%	10%	17%	39%	31%	–	590
Q22.3. Please indicate how often you activate Adaptive Cruise Control in your car. *** [60, 61]	3.83	1.06	6%	6%	15%	47%	27%	–	719
Q36. The surrounding elements (other road users, road borders, marks) detected by my partly automated car are always clear to me. **	3.80	1.04	2%	13%	16%	42%	28%	–	642
Q22.1. Please indicate how often you activate Lane Departure Warning in your car. *** [60, 61]	3.72	1.46	13%	11%	14%	16%	46%	–	719
Q22.2. Please indicate how often you activate Lane Keeping Assist in your car. *** [60, 61]	3.60	1.38	13%	10%	16%	26%	35%	–	719
Q41. I am unwilling to hand over control to my partly automated car from time to time. **	2.86	1.35	22%	23%	14%	30%	11%	–	635
Q59. I am concerned about my general safety most of the time. [38]	2.39	1.21	25%	39%	15%	14%	7%	–	613
Q33. I feel hesitant about activating the partly automated car mode from time to time. [65, 67]	2.38	1.32	33%	29%	11%	19%	8%	–	645
Q34. I engage in other activities while driving my partly automated car. [38, 64, 65]	2.27	1.21	34%	31%	14%	18%	4%	–	643
Q58. I feel bored most of the time. [52]	2.26	0.93	19%	48%	22%	9%	2%	–	609
Q48. I use my partly automated car because it helps me to use my time for other activities unrelated to driving. [38, 51]	2.17	1.15	35%	34%	15%	11%	4%	–	612
Q57. I feel anxious most of the time. [52]	1.94	0.93	36%	44%	13%	6%	2%	–	609

Notes:

* Q26 was measured on a scale from 1 = Never, 2 = Less than monthly, 3 = Less than weekly but more than once a month, 4 = 1–2 times a week, 5 = 3–4 times a week, to 6 = At least 5 times a week.

** Self-created.

** Q22.3 was measured on a scale from 1 = Never, 2 = Rarely, 3 = Occasionally, 4 = Frequently, to 5 = Always.

As shown by Table 2, the highest mean rating was obtained for using the partially automated car with speed and steering assist (***M*** = 4.70, SD = 1.44, on a scale from 1 (never) to 6 (at least five times a week)). The second-highest mean rating was obtained for always knowing when the car is in partially automated driving mode (***M*** = 4.42, SD = 0.87), and the third-highest was obtained for trusting the partially automated car to maintain the speed and distance to the car ahead (***M*** = 4.41, SD = 0.73). The fourth highest rating was obtained for monitoring the performance of the partially automated car most of the time (***M*** = 4.34, SD = 0.87). More than 50% of respondents agreed with these questionnaire items.

The lowest mean rating was obtained for feeling anxious most of the time (***M*** = 1.94, SD = 0.93). The second-lowest and third-lowest ratings were obtained for using the partially automated car for other activities unrelated to driving (***M*** = 2.17, SD = 1.15), and for feeling bored most of the time (***M*** = 2.26, SD = 0.93). The fourth-lowest mean rating was obtained for engaging in other activities while driving the partially automated car (***M*** = 2.27, SD = 1.21).

**Fig 1:**
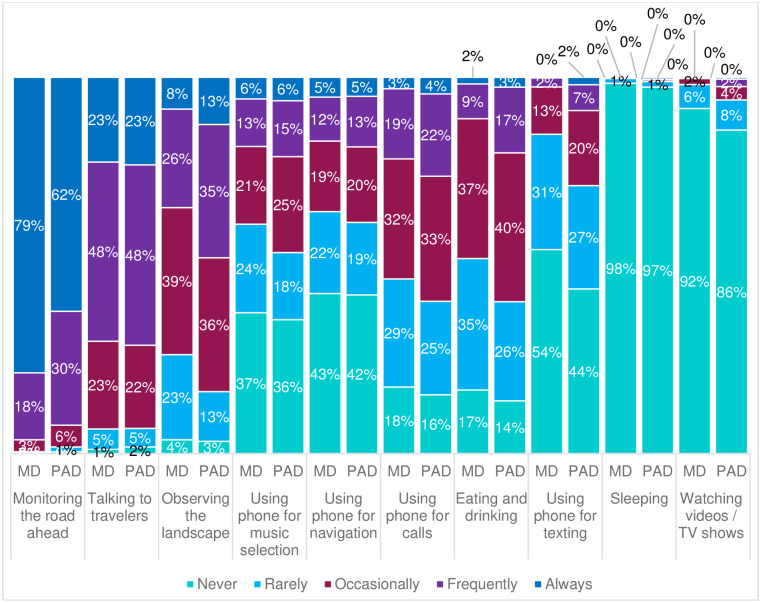
Relative frequencies pertaining to respondents’ engagement in secondary activities during manual driving (MD) and partially automated driving (PAD).

As shown by Fig 1, respondents most frequently engaged in monitoring the road ahead during partially automated driving (PAD) (***M*** = 4.52, SD = 0.71) and manual driving (MD) (***M*** = 4.74, SD = 0.54), followed by talking to fellow travelers (PAD, ***M*** = 3.86, SD = 0.89; MD, ***M*** = 3.85, SD = 0.87), and observing the landscape (PAD, ***M*** = 3.41, SD = 0.98; MD, ***M*** = 3.13, SD = 0.98). Respondents indicated to least frequently engage in watching videos / TV shows (PAD, ***M*** = 1.23, SD = 0.65; MD, ***M*** = 1.11, SD = 0.40), sleeping (PAD, ***M*** = 1.05, SD = 0.35; MD, ***M*** = 1.02, SD = 0.21), and using the phone for texting (PAD, ***M*** = 1.96, SD = 1.04; MD, ***M*** = 1.64, SD = 0.80) during partially and manual automated driving. The proportion of respondents indicating to always monitor the road during manual driving was 79% compared to 62% of respondents reporting to monitor the road during partially automated driving. The results also indicated a modest shift from monitoring the road towards non-driving related activities. Surprisingly, only 1% of respondents (*n* = 8) reported to never monitor the road ahead in PAD. An additional 1% of respondents reported to always (*n* = 3), frequently (*n* = 1), or occasionally (*n* = 3) engage in sleeping during PAD.

### 3.3 Confirmatory factor analysis

**Fig 2:**
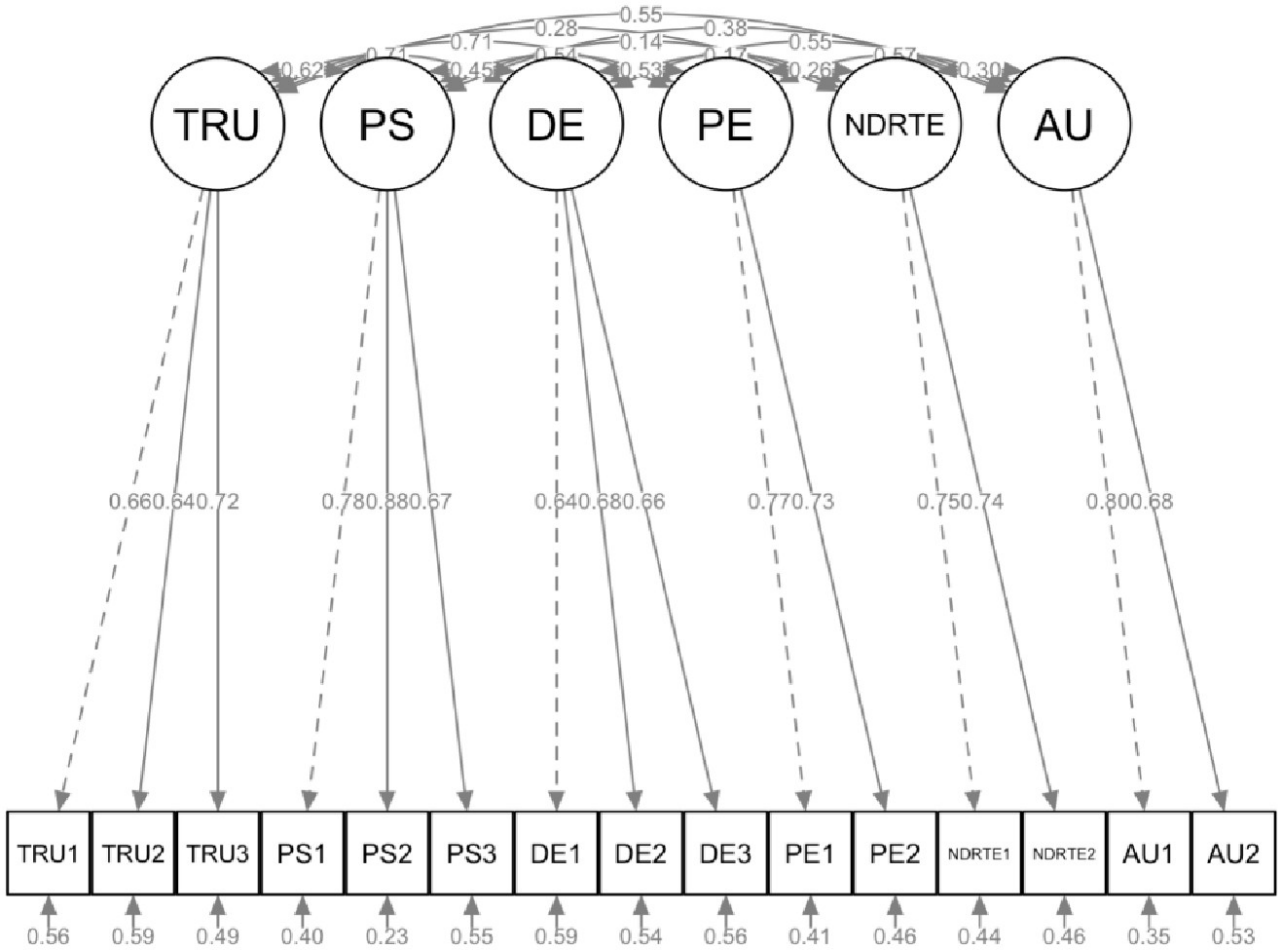
Measurement model. Note that the circles represent the latent (unobserved) constructs; arrows between the latent constructs represent the correlations / covariances between the latent constructs. The boxes represent the observed constructs (questionnaire items). Numbers on the arrows from the latent to the observed constructs represent the lambda’s (i.e., factor loadings). Small arrows underneath the boxes (observed constructs) represent the residuals (i.e., measurement error).

The results of the confirmatory factor analysis are shown in Table 3 and Fig 2. Several items measuring perceived safety (PS4–PS6), trust (TRU3–TRU8), driver engagement (DE4–DE5), performance expectancy (PE3–PE4) were omitted from the analysis as their loading was below the recommended threshold of 0.60. The questionnaire item TRU7 (*“I engage in other activities while driving my partially automated car”*) that did not load strongly enough on trust and the item PE3 *(“I use my partly automated car because it helps me to use my time for other activities unrelated to driving”*) that did not load strongly enough on performance expectancy were merged into the new construct ‘non-driving related task engagement (NDRTE)’ due to their semantic similarity and interpretability. The questionnaire item TRU8 (*“I monitor the performance of my partly automated car most of the time”*) was merged with questions on the construct ‘driver engagement’ due to the interpretability of this item.

**Table 3:** Results of confirmatory factor analysis.

Latent variable	Observed variable	ƛ	α	CR	AVE
**Trust (TRU)**			0.70	0.71	0.45
	TRU1: I trust my partly automated car to maintain speed and distance to the car ahead.	0.66			
	TRU2: I trust my partly automated car to keep the car centered in the lane.	0.64			
	TRU3: I can trust my partly automated car.	0.72			
	TRU4: I feel hesitant about activating the partly automated car mode from time to time (reverse-coded).	Omitted due to factor loading < 0.60			
	TRU5: I am unwilling to hand over control to my partly automated car from time to time (reverse-coded).	Omitted due to factor loading < 0.60			
	TRU6: I always know when my car is in partly automated driving mode.	Omitted due to factor loading < 0.60			
	TRU7: I engage in other activities while driving my partly automated car.	Omitted due to factor loading < 0.60 and merged with question of construct ‘NDRTE’ (non-driving related task engagement)’			
	TRU8: I monitor the performance of my partly automated car most of the time.	Omitted due to factor loading < 0.60 and merged with questions of construct ‘driver engagement’			
**Perceived safety (PS)**			0.81	0.82	0.60
	PS1: I feel safe most of the time.	0.78			
	PS2: I feel relaxed most of the time.	0.88			
	PS3: I feel anxious most of the time (reverse-coded).	0.67			
	PS4: I feel bored most of the time.	Omitted due to factor loading < 0.60			
	PS5: I am concerned about my general safety most of the time (reverse-coded).	Omitted due to factor loading < 0.60			
	PS6: I entrust the safety of a close relative to my partly automated car.	Omitted due to factor loading < 0.60			
**Driver engagement (DE)**			0.70	0.70	0.44
	DE1: My partly automated car always reminds me to take back full control.	0.64			
	DE2: My partly automated car always reminds me to keep my hands on the steering wheel.	0.68			
	DE3: My partly automated car helps me to keep using it in the manner as advised by the manual.	0.66			
	DE4: The surrounding elements (other road users, road borders, marks) detected by my partly automated car are always clear to me.	Omitted due to factor loading < 0.60			
	DE5: I monitor the performance of my partly automated car most of the time.	Omitted due to factor loading < 0.60			
**Non-driving related task engagement (NDRTE)**			0.71	0.71	0.55
	NDRTE1: I engage in other activities while driving my partly automated car.	0.75			
	NDRTE2: I use my partly automated car because it helps me to use my time for other activities unrelated to driving.	0.74			
**Performance expectancy (PE)**			0.72	0.73	0.56
	PE1: I use my partly automated car because it helps me to reach my destination more comfortably.	0.74			
	PE2: I use my partly automated car because it makes driving more pleasurable.	0.77			
	PE3: I use my partly automated car because it helps me to use my time for other activities unrelated to driving.	Omitted due to factor loading < 0.60 and merged with question of construct ‘NDRTE (non-driving related task engagement)’			
	PE4: I use my partly automated car because it helps me to reach my destination more safely.	Omitted due to factor loading < 0.60			
**Automation use (AU)**			0.68	0.71	0.52
	AU1: Please indicate how often you activate ACC in your car.	0.80			
	AU2: Please indicate how often you use your partly automated car with speed and steering support.	0.68			
	AU3: Please indicate how often you activate LKA in your car.	Omitted due to factor loading < 0.60			

Note that ƛ are the factor loadings, which are interpreted as correlation coefficients for the relationship between the questionnaire items and their underlying constructs.

α is the Cronbach’s alpha reliability coefficient, which is a measure for the internal consistency of a latent construct assuming that the correlations between the questionnaire items underlying a latent construct are equal.

Composite reliability is also a measure for the internal consistency of a latent construct, using the varying factor loadings of the questionnaire items on their underlying constructs and their error variance as input for the calculation.

AVE is the average variance extracted that is accounted in the latent construct among the questionnaire items underlying a latent construct.

The fit parameters of the measurement model were acceptable (**C**onfirmatory **F**it **I**ndex (CFI) = 0.93, **R**oot **M**ean **S**quare **E**rror **A**pproximation (RMSEA) = 0.07, and **S**tandardized **R**oot **M**ean **S**quare **R**esidual (SRMR) = 0.05). The chi-square statistic (χ^2^ / *df*) (= 3.31) exceeded the recommended threshold of 3. Composite reliability and Cronbach’s alpha both exceeded the recommended threshold of 0.70 for trust, perceived safety, driver engagement, non-driving related task engagement, and performance expectancy, confirming internal consistency reliability for these constructs. The average variance extracted (AVE) values exceeded the recommended minimum threshold of 0.50 for all constructs except for driver engagement (AVE = 0.44) and trust (AVE = 0.45). As shown by Table 4, which reports the Pearson inter-construct correlations, discriminant validity was acceptable for all latent variables.

**Table 4:** Inter-construct correlation matrix.

**Construct**	**TRU**	**PS**	**DE**	**NDRTE**	**PE**	**AU**
**TRU**	**0.64**					
**PS**	0.45	**0.77**				
**DE**	0.42	0.26	**0.66**			
**NDRTE**	0.21	0.10	0.13	**0.76**		
**PE**	0.50	0.35	0.30	0.19	**0.74**	
**AU**	0.37	0.23	0.38	0.20	0.40	**0.72**

*Note*: The diagonal values represent the square root of the average variance extracted (AVE).

### 3.4 Structural equation modeling analysis

**Fig 3:**
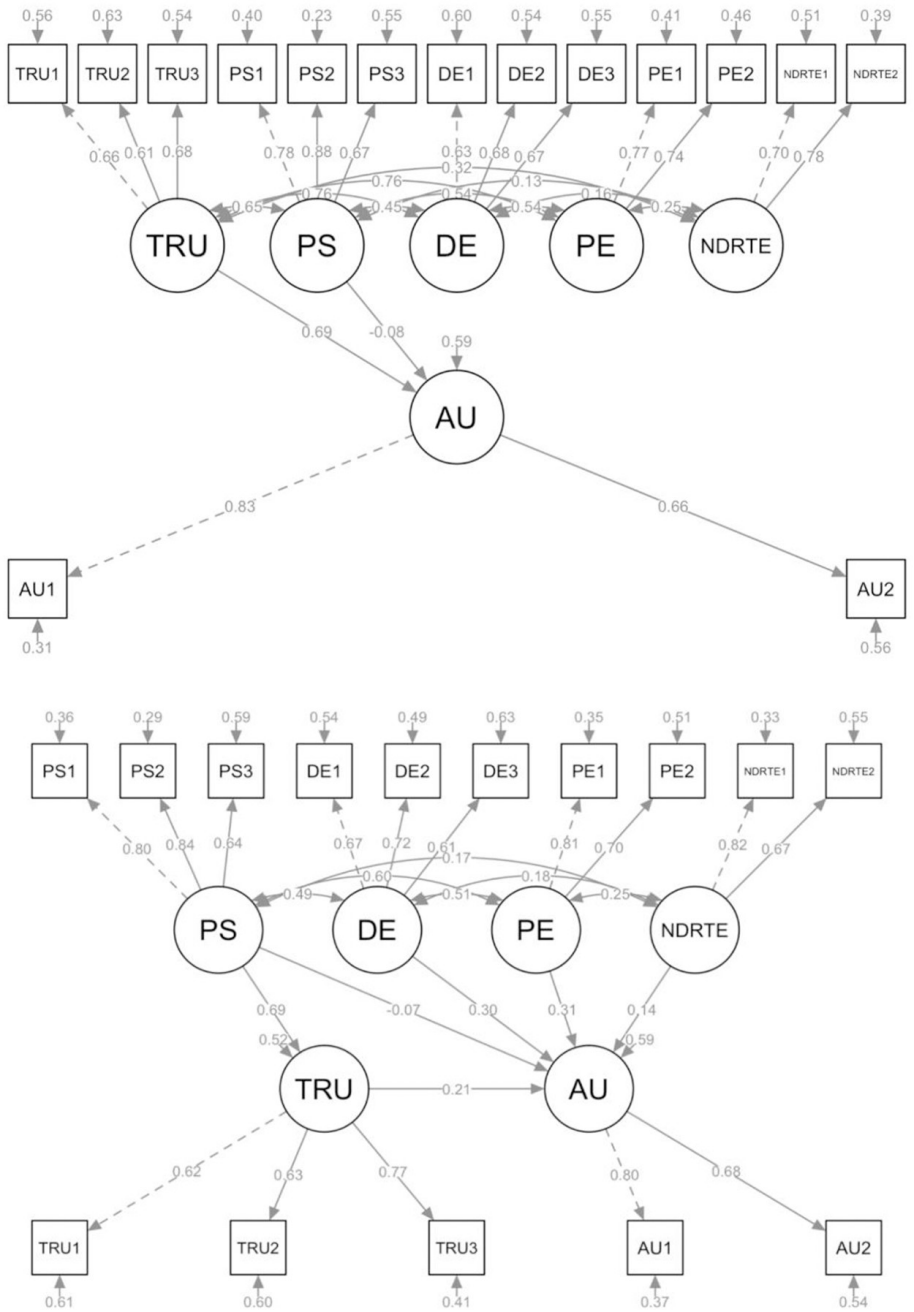
Structural model a and b.

We analyzed two structural models capturing the relationship between our study constructs. In the first model, perceived safety and trust were identified as predictors of automation use. As shown by Fig 3a, the relationship between trust and automation use was significant (*β* = 0.69, *p* = 0.001). Trust explained 41.3% of the variance in behavioral intention. Perceived safety did not influence automation use directly (*β* = -0.08, *p* = 0.29). As the direct effect of perceived safety on automation use was negative and not significant, we tested whether trust mediated the relationship between perceived safety and automation use in a second structural model (Fig 3b). In addition, we added the predictors driver engagement, non-driving related task engagement, and performance expectancy to the model. The analysis revealed that performance expectancy had the strongest effect on automation use (*β* = 0.31, *p* = 0.001), followed by driver engagement (*β* = 0.30, *p* = 0.001), and non-driving related task engagement (*β* = 0.14, *p* = 0.01). Trust mediated the relationship between perceived safety and automation use. The path from trust to automation use was positive and significant (*β* = 0.21, *p* = 0.02). Perceived safety had significant positive effects on trust (*β* = 0.69, *p* = 0.001). As in the first model, perceived safety did not predict automation use directly (*β* = -0.07, *p* = 0.46). The variance explained in automation use was still 41.3%, meaning that the addition of the other predictor variables did not increase the explanatory power of the model.

## 4. Discussion

The present study surveyed drivers of partially automated cars to address the following research questions.

### 4.1 Research question 1: What are the activities that drivers of partially automated cars engage in during manual and partially automated driving?

One of the lowest mean ratings was obtained for engaging in other activities while driving the partially automated car (***M*** = 2.27). Respondents indicated that they most frequently monitored the road ahead, talked to fellow travellers, and observed the landscape, while they least frequently watched videos or TV shows, slept, or used the phone for texting during partially automated driving. Our respondents seemed to take their monitoring obligations during partially automated driving seriously, reporting to monitor the performance of their car most of the time (***M*** = 4.34). These findings stand in contrast to the studies and videos showing inappropriate use of Tesla’s Autopilot system (e.g., prolonged hands-free driving, ignoring warnings to place hands back on the steering wheel, testing the limits of the operational design domain, mode confusion, engagement in secondary activities, using the system in bad weather conditions, using the system not on highways, misleading the hand detection by attaching objects to the steering wheel, leaving the driver seat, falling asleep) [4–8, 68–73]. Partial automation did, however, increase reported engagement in secondary tasks that are already performed during manual driving (i.e., the proportion of respondents reporting to observe the landscape, use the phone for texting, navigation, music selection and calls, and eat during partially automated driving was higher in comparison to manual driving). Unsafe behaviour (automation misuse) was hardly reported as only 1% of respondents indicated to rarely monitor the road while using partially automated driving, and another 1% of respondents reported to always, frequently, or occasionally sleep during partially automated driving. However, such rare behaviours can still lead to a relevant number of accidents. Note that there is a paucity of scientific studies with real-world SAE Level 2 passenger cars [70, 74–76]. Therefore, it is not clear to what extent these unsafe behaviors of drivers of partially automated cars represent long- or short-term effects of automation, and why these behaviors actually occur. It is also plausible that some drivers believed that taking their hands off the wheel and watching a video was safe [77], or that some staged falling asleep in order to contribute to the hype around Tesla’s Autopilot system.

### 4.2 Research question 2: How are the perceived safety and trust in partially automated cars operationalized?

Previous studies on road vehicle automation have operationalized perceived safety and trust by generic items, such as: *“Overall*, *AVs would help make my journeys safer than they are when I use conventional vehicles”* [78, p. 3; 79, p. 874], *“I am worried that the general safety of using an AV is worse than that of driving a common vehicle”* [80, p. 109848], and *“Overall*, *I can trust autonomous vehicles”* [29, p. 697]. Other studies asked respondents to rate their level of trust and safety or changes in these using items such as: *“To what extent do you trust the driving automation according to the previous performance of the system*?*”* [81], *“Ranked the buttons on safety perception scale”*, [82, p. 351], and *“Please indicate the degree that your trust has changed after this encounter”* [83]. These items were not tailored to the specific nature of partial automation requiring permanent supervision by human drivers. Trusting in partially automated driving system was tested for parking (*“To what extent do you trust the Tesla’s ability to park itself*?*”)* [84, p. 197]. The present study contributed to the development of scales to measure trust and perceived safety in partially automated cars. The confirmatory factor analysis revealed that trust mainly depended on longitudinal automation performance (*“TRU1*: *I trust my partly automated car to maintain speed and distance to the car ahead”*), lateral performance (*“TRU2*: *I trust my partly automated car to keep the car centered in the lane”*), and overall trust (*“TRU3*: *I can trust my partly automated car”*). The self-developed items *“TRU4*: *I feel hesitant about activating the partially automated car mode from time to time”* (reverse-coded), *“TRU5*: *I am unwilling to hand over control to my partially automated car from time to time”* (reverse-coded) and *“TRU8*: *I monitor the performance of my partially automated car most of the time”* were dropped as their loadings on trust were insufficient. These items imply an evaluation of the frequency (i.e., from time to time, most of the time) of situational usage behaviour (i.e., activation of partially automated driving, handing over control to the car, and driver monitoring). Our results do not support usage of such items to assess trust in partial automation. This finding also points to a distinction between general trust (*“TRU3*: *I can trust my partly automated car”*) and behavioural trust in partially automated driving (*“TRU4*: *I* ***feel hesitant about activating*** *the partially automated car mode from time to time”*, *“TRU5*: *I am unwilling to* ***hand over control*** *to my partially automated car from time to time”* and *“TRU8*: *I* ***monitor the performance*** *of my partially automated car most of the time”*. Our findings indicate that respondents have an accurate understanding of where, when and how to use their partially automated cars in order to trust it. This supports the notion that trust in automation is inherently context- / situation- specific (e.g., *“I trust the automation in this situation”*) [85, p. 41] and differs across driving scenarios [86]. The items *“TRU6*: *I always know when my car is in partially automated driving mode”* and *“TRU7*: *I engage in other activities while driving my partially automated car”*, which were based on the literature, were excluded as indicators of trust. This is plausible as the formulation of these items is very specific and tailored to aspects that may be conceptually unrelated to trust such as non-driving related task engagement and driver engagement. In partially automated cars, drivers are not allowed to engage in non-driving related activities. Furthermore, drivers need to know the mode the partially automated car is in regardless of trust since it dictates task distribution.

Perceived safety was measured by three items established from the literature. The item *“PS2*: *I feel relaxed most of the time”* had the strongest loading on perceived safety, indicating that feelings of relaxation may be most decisive for feelings of perceived safety in partially automated cars. While the item *“PS1*: *I feel safe most of the time”* had the second-strongest loading on perceived safety, the reversely coded item *“PS5*: *I am concerned about my general safety most of the time”* did not load sufficiently. The item *“PS3*: *I feel anxious most of the time”* (reverse-coded) had the third-strongest loading on perceived safety. This indicates that perceptions of safety in partially automated cars are strongly associated with emotional and affective dimensions. Others [87, p. 4] validated a scale for perceived safety for intelligent connected vehicles (ICVs), measuring cognitive components (e.g., *“I think the potential danger of an ICV is acceptable”)* and emotional components of safety (e.g., *“I think it’s relaxing to operate an ICV”)*. The item *“PS4*: *I feel bored most of the time”* was also omitted from the analysis, meaning that feeling bored was not associated with perceptions of safety in the context of partial automation. The evaluation of the acceptance of SAE Level 4 driverless shuttles using data from respondents who physically experienced automated shuttles resulted in the distinction between the theoretical constructs perceived safety and boredom [52]. The questionnaire item *“PS6*: *I entrust the safety of a close relative to my partially automated car”* was also omitted from the scale perceived safety. This suggests that feelings of perceived safety are more oriented towards the individual rather than close relatives. Future studies should test whether the questions that were not included as valid and reliable indictors of trust and perceived safety in the present study, respectively, can become so in SAE Level 2+ vehicles, taking into account the corresponding role of human drivers.

### 4.3 Research question 3: To what extent do drivers perceive their partially automated cars safe and trustworthy?

Respondents rated the perceived safety and trust while using their partially automated cars as very high. Over 80% of respondents indicated to feel safe and relaxed most of the time, while only 8% reported to have feelings of anxiety during partially automated driving. This is in line with reports from manufacturers claiming that their partially automated cars are indeed safer (than manual driving) [88]. Over 80% of respondents testing different L3 conditionally automated driving functions in the context of the L3Pilot project indicated to feel safe when driving with the system active, and more than 60% indicated to feel safe in take-over situations [89]. The assumption that partially automated driving is safer than manual driving may hold if the car is used appropriately. Note, however, that a formal assessment of automation effects on safety would require substantially more data than is currently available [90]. Regarding their ratings of trust, 89% of respondents reported to trust their partially automated car maintaining the speed and distance to the car ahead. Over 70% agreed with the statements to trust their partially automated car, and to trust their partially automated car keeping the car centered in the lane. This matches a survey [70] where 90% of respondents considered the partially automated driving system dependable and 78% of respondents reported to trust it. Our respondents seemed to have a solid understanding of the car’s capabilities and limitations: A high mean rating (***M*** = 4.02) was obtained for the partially automated car helping drivers to use it as advised by the manual. This suggests that respondents were aware of the car’s capabilities and limitations as well as of their role as driver. Other studies have shown inaccurate expectations of the capabilities of partially automated cars [86]. In [91], 57% of respondents reported to know “very little” and 23% of respondents “a moderate amount” of autonomous vehicles. In our recent study [61] with 18,631 respondents from 17 countries, respondents were inaccurate about the operation of conditionally automated cars (SAE level 3) being limited to operational design domains. Furthermore, only 5% and 8% of respondents from the Dominican Republic knew Intelligent Transportation Systems (ITS) in 2018 and 2019, respectively [92]. One plausible explanation for our positive finding regarding understanding automation is that our respondents were experienced drivers of partially automated cars.

### 4.4 Research question 4: How are perceived safety and trust in partially automated cars related?

Structural equation modeling revealed a positive relationship between perceived safety and trust (*β* = 0.69, *p* = 0.001), which corresponds with other studies [38, 52, 81]. Our finding suggests that individuals who provided higher ratings to the safety of their partially automated cars were more likely to consider partially automated cars as trustworthy than individuals who provided lower ratings to perceived safety. This matches various studies showing positive effects of perceived safety on trust [50, 52, 93]. The finding implies that increasing the perceived safety of partially automated cars is a useful avenue to promote trust in partially automated cars.

### 4.5 Research question 5: How do performance expectancy, perceived safety, trust, driver engagement, and non-driving related task engagement relate with the acceptance of partially automated cars?

In the second structural model, performance expectancy had the strongest effect on automation use (*β* = 0.31, *p* = 0.001), followed by driver engagement (*β* = 0.30, *p* = 0.001), trust (*β* = 0.21, *p* = 0.02), and non-driving related task engagement (*β* = 0.14, *p* = 0.01). The intention to use automated vehicles was strongly related to performance expectancy and the perceived benefits of automated cars [38, 50, 51, 79, 80, 93, 94]. This suggests that individuals who appreciated the benefits of (partially) automated cars are more likely to form positive intentions to use these cars. Driver engagement, trust in partially automated cars, and non-driving related task engagement were the second, third, and fourth strongest predictors of automation use, respectively. This suggests that keeping the driver engaged in the driving task, promoting trust in partially automated cars, and encouraging engagement in non-driving related activities can be useful ways to promote the use of partially automated cars. We recommend future research to revisit our scale measuring driver engagement as this is pivotal in SAE Level 2–4 cars. The relationship between perceived safety and automation use was not significant in both structural models. This is in contrast to research studies showing positive effects of perceived safety on the intention to use automated vehicles [38, 79]. However, positive effects of perceived safety on trust were found, indicating that the effect of perceived safety on automation was mediated by trust, which is in line with other studies [93].

### 4.6 Limitations and implications for future research

First, respondents may not necessarily be representative of the general population of drivers of partially automated cars. The interest in, knowledge about, and enthusiasm for this technology may be higher among our respondents compared to the general population, possibly because the majority of respondents were recruited from platforms attracting people with a high interest in automated vehicles. On the other hand, it could be argued that most previous studies with partially automated cars addressed expected behaviour in future partially automated cars not yet experienced by respondents. The present study evaluated actual experienced drivers of partial automation, documenting adequate understanding and behaviour.

Second, respondents reporting safe behaviour may not always use automation safely. They may have answered questions in a socially desirable way given their awareness of the misuse of Tesla’s Autopilot system. Future work should investigate to what extent partially automated driving encourages risky driving in comparison to manual driving through observations of actual behaviour in naturalistic driving settings, and analysis of real-world accident statistics.

Third, the causality of the relationship between perceived safety and trust can’t be proven due to the cross-sectional nature of the survey data. We recommend future research to examine the nature of the relationship between perceived safety and trust. That is, do drivers feel safe because they trust partially automated cars, or do they trust partially automated cars because they feel safe? Is this relationship of a correlational rather than causal nature? This can be pursued studying the development of perceived safety and trust in time and across conditions of varying automation performance and criticality of driving conditions.

Fourth, it should be noted that respondents may find it difficult to clearly discriminate between the constructs of perceived safety and trust in survey research as it is likely that respondents attach a similar meaning to these constructs. We recommend future research to use neuro-physiological, objective data (e.g., number of manual interventions, eye glance behaviour, heart rate frequency) [74, 84, 95–97], and link these with the subjective self-reported measures of perceived safety and trust.

## 5 Conclusions

Respondents reported high levels of perceived safety and trust in their partially automated cars. We also found high mean ratings for always knowing when the car is in partially automated mode, and for monitoring the performance of the partially automated car most of the time. One of the lowest mean ratings was obtained for engaging in secondary activities while driving the partially automated car. Unsafe behaviour was rare with 1% reporting to rarely monitor the road and 1% reporting to sleep in their partially automated cars. Structural equation modeling analysis revealed positive effects of perceived safety on trust. Perceived safety did not directly influence automation use but interacted with automation use through trust. Trust significantly affected automation use in addition to performance expectancy, driver engagement and non-driving related task engagement. The present study contributed to the development of scales for trust, perceived safety, driver engagement, and non-driving related task engagement.

## Supporting information

S1 Data (XLSX)
